# Frequency, Risk Factors, and Mortality for Diabetes Mellitus in 1 225 130 Cats Under Primary Veterinary Care in the United Kingdom in 2019

**DOI:** 10.1111/jvim.70161

**Published:** 2025-06-17

**Authors:** Oliver Waite, Ruth Gostelow, Emma Wright, Rosanne E. Jepson, Dave C. Brodbelt, Dan G. O'Neill

**Affiliations:** ^1^ Department of Clinical Science and Services, Royal Veterinary College University of London Hertfordshire UK; ^2^ Murdoch University, School of Veterinary Medicine Murdoch Australia; ^3^ Pathobiology and Population Sciences, Royal Veterinary College University of London Hertfordshire UK

**Keywords:** body weight, diabetic, epidemiology, first opinion veterinary care, purebred, VetCompass

## Abstract

**Background:**

Diabetes mellitus (DM) is a common endocrinopathy in cats. General population epidemiology and mortality studies on DM are lacking.

**Objectives:**

Describe the incidence, prevalence, risk factors, and mortality for DM in cats under primary veterinary care in the United Kingdom (UK).

**Animals:**

Random sample of 1053 confirmed DM cases from 1 255 130 cats in VetCompass.

**Methods:**

Retrospective cohort study with a nested case–control study. Period prevalence and incidence risk of DM for 2019 were calculated. Multivariable logistic regression modeling was used to identify risk factors for DM.

**Results:**

Annual prevalence was 0.39% (95% confidence interval [CI]: 0.37–0.42). Incidence risk (2019) was 0.14% (95% CI, 0.13–0.16). Mean age and median adult body weight of incident cases diagnosed with DM was 11.8 ± 3.5 years (*n* = 371) and 5.9 kg (interquartile range: 4.6–7.1, *n* = 327). Odds of DM were increased in cats ≥ 9.0 years (odds ratio [OR]: 5.11, CI, 4.10–6.24) compared with cats 4.5–9.0 years. Burmese (OR: 2.07, CI, 1.29–3.31) and Burmillas (OR: 8.30, CI, 2.59–26.62) had increased odds, whereas Bengals (OR: 0.24, CI: 0.06–0.98) and Ragdolls (OR: 0.11, CI: 0.02–0.80) had decreased odds of DM compared with crossbreeds. Of 51.2% (192/375) cats dead within 3 years of diagnosis, 93.0% (176/192) were euthanized; 19.7% (35/178) were euthanized ≤ 3 days after diagnosis.

**Conclusions and Clinical Importance:**

Almost 1/250 cats in the UK live with DM annually. Burmillas were predisposed to DM, and protection against DM was shown in Bengals and Ragdolls. Early mortality associated with DM diagnosis in cats is high.

**Abbreviations**
CI: confidence interval
DM: diabetes mellitus
EHR: electronic health record
IQR: interquartile range
OR: odds ratio
PZI: protamine zinc insulin
QoL: quality of life
SGLT‐2: sodium–glucose cotransporter‐2
UK: United Kingdom

## 1 Introduction

Diabetes mellitus (DM) is a common endocrinopathy in cats [1]. The reported United Kingdom (UK) prevalence of DM in cats ranges between 0.43% and 0.58% [2, 3]. Internationally, the prevalence of cats living with DM ranges between 0.21% and 0.74% based on insurance and veterinary practice population data [4, 5].

The incidence risk for newly diagnosed cases of DM in cats is poorly defined and has not been described in cats under primary veterinary care.

Adult body weight, age, breed, neuter status, and sex are risk factors for DM in cats that have been extensively investigated [2, 3, 6, 7, 8, 9, 10, 11], but results originate from insured or referred populations or both, which may limit generalizability to the wider population [3, 4, 8, 9, 11, 12]. Moreover, breed and neuter status are reported risk factors with confounding results [13, 14].

Despite crossbreeds being reported at an increased risk of DM compared with purebred cats, increased odds of DM are reported in certain purebreds including European and Australian Burmese, Norwegian Forest, and Tonkinese [2, 3, 5, 6, 8, 15, 16], and although protection against DM is postulated in the Birman [2, 16], robust evidence describing individual purebred cats with a decreased risk of DM is lacking.

Previous studies investigating the epidemiology and survival of cats with DM under UK primary veterinary care are now considered dated, describing results before the introduction of now commonplace treatments such as protamine zinc insulin (PZI) [2, 17].

Mortality rates among cats after DM diagnosis are reportedly high, but these data are also now considered dated. Previous studies have reported 30‐day and 1‐year mortality rates up to 17.0% and 41.8%, respectively [18, 19, 20, 21]. Factors driving early mortality remain poorly defined, and owner reluctance to pursue management is one probable contributing factor [20, 21]. Recent licensing of orally administered once daily sodium–glucose cotransporter‐2 (SGLT‐2) inhibitors in the UK now offers an alternative and potentially easier management strategy for cats with DM [22, 23, 24]. Although future mortality information following the advent and use of SGLT‐2 inhibitors will prove valuable, DM mortality with traditional treatment options including PZI still warrants review.

We aimed to generate updated epidemiological and risk factor evidence on DM in cats under UK primary veterinary care practice. A further aim of the study was to describe mortality in cats after diagnosis of DM.

## 2 Methods

### 2.1 Case Selection

A retrospective cohort study was designed using data from all cats under UK primary veterinary care at practices participating in the VetCompass program with at least one electronic health record (EHR) during 2019. The VetCompass program extracts anonymized EHR data from participating practice management systems using integrated clinical queries that are uploaded to a secure VetCompass structured query language database [2]. All available EHRs from all available dates within the practice management systems are uploaded to VetCompass, with the earliest available year being 2012 soon after VetCompass data collection began. Veterinarians are not required to code diagnoses or change their preferred note‐making style. Instead, routinely recorded notes and other clinical information are shared and later examined by VetCompass researchers to extract information pertaining to specific research questions [25]. Researchers can access patient signalment (species, breed, date of birth, sex, neuter status) and clinical data (free clinical text, treatment fields and body weight) between relevant dates to answer specific research questions [2].

Based on prior evidence for a 0.58% prevalence of DM in cats under primary veterinary care in the UK [2], a power calculation estimated that a study sample of 21 714 cats was needed to estimate prevalence with a 0.1% acceptable margin of error at a 95% confidence level from a national UK population of 11 million cats [26]. Ethical approval was obtained from the Royal Veterinary College Ethics and Welfare Committee (reference number SR2018‐1652) before commencing data retrieval.

The study design, data extraction, and analysis methods were designed to align with previously published VetCompass studies to facilitate inter‐study comparisons [2]. Estimates of the 2019 incidence risk and 1‐year (2019) period prevalence of DM in cats under UK primary veterinary care were calculated by reviewing EHR data from cats under UK primary veterinary care in 2019. Cats under UK primary veterinary care in 2019 were defined as those possessing an EHR entry for a cat alive any time during 2019. Data were available for review from the first available EHR entry: May 14, 2012, until the end of the follow‐up period: December 31, 2021. Automated searching of EHR data used terms to identify candidate cases (potential cases of DM in cats) from clinical notes: (diab*, diab* + keto*, DKA, fruct*, insulin) and from the treatment fields (caninsulin, diab*, glargine, glipizide, hypurin, insulin, neutral ins*, prozinc, vetpen). This list of search terms relevant to the diagnosis and management of DM in cats was developed and iteratively refined to optimize the positive and negative predictive power to either rule in or rule out the candidate cases as being confirmed cases.

A random sample of candidate cases was manually reviewed to confirm or exclude a final diagnosis of DM. A DM case was defined as any cat with DM, recorded as the final diagnosis (or synonym) in the EHR at any date up until December 31, 2019. Exclusion criteria included cats where a DM diagnosis was only listed as an option within a longer list of potential diagnoses, or where DM was speculated earlier in the EHR but was later ruled out. All cats with evidence of DM at any time point during and until the end of the study period (December 31, 2019) were recorded and defined as prevalent DM cases. For each cat that was confirmed as being a DM case during 2019, all available clinical records for that cat within VetCompass were manually reviewed to identify the date of the first lifetime diagnosis of DM. Cats with a confirmed first lifetime diagnosis of DM occurring January 1 to December 31, 2019 were defined as incident cases and the EHRs of these cats were followed until the end of the follow‐up period: December 31, 2021. Cases that were first diagnosed with DM before January 1, 2019 were considered pre‐existing, and these cases were added to the incident cases to generate the overall prevalent cases for 2019. Clinical data pertaining to whether or not anti‐hyperglycemic treatment was administered, as well as the type of antihyperglycemic treatment used, was collected but not used in the current epidemiological study. These data instead will be included in a future study that will explore clinical management in more detail.

### 2.2 Risk Factors for Diabetes Mellitus

Risk factor analysis used a nested case–control design. A random sample of non‐cases was included as controls rather than including all non‐cases to facilitate the data preparation required for the large number of overall non‐cases. Signalment was extracted automatically from the VetCompass database for all cats and compared between cats with a confirmed diagnosis of DM within the 2019 sampling frame (prevalent cases) and a random sample of 500 000 non‐cases to investigate risk factors associated with a diagnosis of DM. Non‐cases were defined as cats under UK primary care practice in 2019 that were not identified as candidate DM cases and therefore did not contain any of the listed search terms within their EHR.

Breed descriptive information entered by participating veterinary practices was cleaned and mapped to a VetCompass breed list derived and extended from the VeNom coding breed list [27]. Cats where no breed information was available were defined as ‘unrecorded’. A breed variable included individual breeds with ≥ 2000 animals in the overall VetCompass dataset of cats under veterinary care in 2019 which comprised both DM cases (prevalent) and non‐cases. Breeds with 1000–2000 individual purebred animals in the overall dataset were grouped under the title “other purebreds” (*n* = 11 879). This approach was taken to facilitate statistical power for the individual breed analyses.

Purebred cat breeds with < 1000 individual animals in the overall dataset were excluded from final breed analysis because of the low numbers of individual animals in each individual breed. The number of individual purebred animals within this group of excluded animals (i.e., < 1000 individual animals in each of the purebred breeds) was low (*n* = 9185).

A purebred variable categorized cats of recognizable breeds as “purebred” whereas cats recorded as mixes between breeds were described as “crossbreed” [28]. Age (years) recorded at December 31, 2019 was calculated for each cat diagnosed with DM at any time point in the study period (prevalent case) and was categorized into six groups: ≤ 4.5, 4.5–9.0, 9.0–13.5, 13.5–18.0, ≥ 18.0, or unrecorded. The median adult body weight (≥ 9 months of age in kg) for each cat diagnosed with DM at any time point in the study period (prevalent case) was categorized into eight groups: ≤ 2.9, 3.0–3.9, 4.0–4.9, 5.0–5.9, 6.0–6.9, 7.0–7.9, ≥ 8.0, or unrecorded. Age and adult body weight data from cats dead before the end of the study period (December 31, 2019) were included in the final analysis, using the last available data from the EHR. Data regarding sex and neuter status were automatically extracted from EHR of cats with DM in 2019 (prevalent cases) using the VetCompass program.

The EHRs were reviewed for information describing cats as overweight (or synonym) any time within a 1‐year period before an incident (2019) first lifetime diagnosis of DM. EHRs additionally were reviewed for body composition information in cats with an incident (2019) first lifetime diagnosis of DM recorded within 7 days before or after diagnosis. This information was categorized using EHR clinical text entries as: not stated, underweight severity not specified, underweight severe, underweight mild, normal, overweight severity not specified, overweight mild, and overweight obese.

Finally, EHR data were reviewed for data pertaining to concurrent disease at the time of DM diagnosis. Diagnosis of hyperthyroidism and chronic kidney disease were identified by review of the EHR and was categorized as either pre‐existing or concurrently diagnosed alongside DM.

### 2.3 Mortality

Cats with an incident (2019) diagnosis of DM while under UK primary veterinary care were followed throughout the follow‐up period until whichever of the following events occurred first: death, lost to follow‐up, or end of the follow‐up period (December 31, 2021). For each death, information was manually extracted regarding the date and type of death (euthanasia, natural, or unrecorded). The biomedical cause of any death was further described as directly attributable to DM, partially attributable to DM (i.e., DM was a contributing factor), unrelated to DM, or unrecorded. Cats euthanized ≤ 3 and ≤ 30 days after a diagnosis of DM were recorded as such. Our decision to report the ≤ 3 day mortality rate after an incident (2019) DM diagnosis evolved after review of EHR; many cats euthanized soon after DM diagnosis (≤ 3 days) had not received any antihyperglycemic treatment.

### 2.4 Statistical Analysis

After data checking and cleaning in Excel (Microsoft Office Excel 2018, Microsoft Corp.), analyses were conducted using IBM SPSS Statistics for Macintosh, Version 27.0. Armonk, NY: IBM Corp. Survival analyses were performed in GraphPad Prism version 10.0.2 (171).

One year period prevalence and incidence risk, both for 2019, described the probability of a prevalent or incident diagnosis during 2019 for cats under UK primary veterinary care. Because the sampling design involved verification of a subset of candidate cases, the estimated total number of prevalent or incident cases in the overall dataset was calculated by scaling up from the proportion of the checked candidate cases that were confirmed as cases to estimate the number that would have been confirmed if all candidate cases were checked. Estimates of confidence interval (CI) were derived from standard errors based on approximation to the binomial distribution [26].

The mean and SD were reported for normally distributed data, and the median, interquartile range (IQR) and range were reported for non‐normally distributed data [29]. The mean age at diagnosis across incident (2019) diabetic cats was calculated using the date of birth and date of diagnosis data available in the patient EHR. The adult body weight of cats at the time of an incident (2019) diagnosis of DM was recorded using data available in the EHR, up to 1 week on either side of the first lifetime DM diagnosis date. The frequency (%) of sex and neuter status were recorded for all cats with a diagnosis of DM while under UK primary care practice in 2019 (prevalent cases).

Risk factor analysis used multivariable binary logistic regression modeling to compare all cats with a verified diagnosis of DM while under UK primary veterinary care practice in 2019 (prevalent cases) to a random sample of 500 000 non‐cases. First, univariable logistic regression modeling explored associations between risk factors (breed, purebred, age, adult body weight, sex, and neuter status) and being a DM case. Risk factors in the univariable modeling with *p* < 0.2 were carried forward for multivariable evaluation, using automated backwards selection. Model fit in the final multivariable model was assessed using the Hosmer‐Lemeshow test. The models were not assessed for interactions.

Breed was a factor of primary interest and thus the main multivariable model contained the breed variable. The breed variable was later replaced by the purebred variable to explore the association between purebred status and DM diagnosis. For the final breed‐based model, the area under the receiver operating characteristic curve was used to evaluate the quality of the model fit. The Kaplan–Meier method was used to calculate survival curves from observed survival times in cats after an incident (2019) DM diagnosis. Statistical significance was set at *p* < 0.05.

## 3 Results

### 3.1 Descriptive Results

From a study cohort of 1 255 130 cats under UK primary veterinary care at 1224 clinics within six veterinary groups in 2019, 14 321 (1.1%) candidate DM cases were identified. Manual review of a random sample of 3047/14 321 (21.3%) candidate DM cases confirmed 1053 to be prevalent DM cases. As described in the methods, this estimates that 4944 prevalent DM cases would have been identified in all 14 321 of the candidate cases had they been manually examined. The 1‐year period prevalence of DM in cats under UK primary veterinary care in 2019 was 0.39% (95% CI, 0.37–0.42). The 1053 confirmed prevalent cases consisted of 678 (64.4%) cats first diagnosed before January 1, 2019 and 375 (35.6%) incident (2019) cases. The 1‐year (2019) incidence risk for DM in cats was 0.14% (95% CI, 0.13–0.16).

Prevalent cases consisted of 665 (69.3%) male and 380 (36.1%) female cats. Sex was not recorded in eight (0.8%) cats. Eight hundred fifty‐two (81.0%) prevalent diabetic cats were neutered, whereas 192 (18.2%) cats were recorded as intact. The date of DM diagnosis was unavailable in 27.1% (285/1053) of overall (prevalent) DM cases.

The age at first diagnosis of incident (2019) diabetic cats with information available was 11.8 ± 3.5 years (*n* = 371 [98.9%]).

The adult body weight of 2019 incident diabetic cats was 5.9 kg (IQR: 4.6–7.1, range: 1.5–14.5 kg, *n* = 327). Seventy‐nine (21.1%) incident cases were described as overweight (or synonym) within the 1‐year period before the first DM diagnosis. Body composition information was available for 105 (28.0%) incident (2019) cases; 28 (26.7%) cats were described as overweight and 56 (53.3%) cats were described as underweight (or synonym).

Among prevalent cases, there were 967 (91.8%) crossbreed cats, 79 (7.5%) purebred cats, and seven (0.7%) cats where breed information was unavailable. The breeds that contributed most to the cases in the overall data set were: Burmese (18/1053, 1.7%), British Short Hair (14/1053, 1.3%), Maine Coon (13/1053, 1.2%), Siamese (6/1053, 0.6%), and Persian (4/1053, 0.4%). Ten (1.0%) purebred cats with a DM diagnosis in 2019 (prevalent cases) were listed under “other purebreds” and further information describing crossbreed and purebred cats, including individual purebreds, is detailed in Table 1.

**TABLE 1:** Descriptive and univariable logistic‐regression results in cats in the VetCompass database with a prevalent (2019) diagnosis of diabetes mellitus while under UK primary veterinary care: *n* = 1053 and cats under UK primary veterinary care (2019) without DM: *n* = 1 254 077.

Variable	Category	DM case no. (%)	Non‐case no. (%)	Odds ratio	95% Confidence interval	Category *p*	Variable *p*
Purebred status							< 0.001
	Crossbreed	967 (91.8)	1 065 863 (87.0)^a^	Base			
	Purebred	79 (7.5)	143 340 (11.7)	0.60	0.48–0.76	< 0.001	
	Unrecorded	7 (0.7)	15 927 (1.3)	0.37	0.18–0.78	0.01	
Breed							< 0.001
	Crossbreed	967 (91.8)	1 087 915 (88.8)^a^	Base			
	Abyssinian	3 (0.3)	1103 (0.09)	2.90	0.93–9.04	0.07	
	Bengal	2 (0.2)	15 192 (1.2)	0.15	0.37–0.59	0.01	
	British Short Hair	14 (1.3)	36 754 (3.0)	0.43	0.25–0.72	0.002	
	Burmese	18 (1.7)	6126 (0.5)	3.48	2.18–5.56	< 0.001	
	Burmilla	3 (0.3)	368 (0.03)	10.3	3.28–32.51	< 0.001	
	Maine Coon	13 (1.2)	12 251 (1.0)	1.20	0.69–2.07	0.50	
	Norwegian Forest Cat	2 (0.2)	2450 (0.2)	0.89	0.22–3.55	0.90	
	Other purebreds	10 (1.0)	3675 (0.3)	0.63	0.26–1.51	0.30	
	Persian	4 (0.4)	9801 (0.8)	0.43	0.16–1.13	0.09	
	Ragdoll	1 (0.1)	20 827 (1.7)	0.53	0.01–0.37	0.003	
	Russian Blue	2 (0.2)	3675 (0.3)	0.64	0.16–2.58	0.50	
	Siberian	1 (0.1)	2450 (0.2)	0.50	0.50–0.70	0.50	
	Siamese	6 (0.6)	8576 (0.7)	0.75	0.34–1.67	0.50	
	Sphinx	0 (0.0)	3675 (0.3)	0.00	0.00	1.0	
	Unrecorded	7 (0.7)	15 927 (1.30)	0.38	0.22–0.67	< 0.001	
Adult (≥ 9 month bodyweight [kg])							< 0.001
	4.0–4.9	149 (14.2)	127 414 (10.4)	Base			
	≤ 2.9	45 (4.3)	75 958 (6.2)	0.50	0.35–0.69	< 0.001	
	3.0–3.9	89 (8.5)	105 361 (8.6)	0.76	0.58–1.00	0.05	
	5.0–5.9	178 (16.9)	120 063 (9.8)	1.27	1.01–1.60	0.04	
	6.0–6.9	181 (17.2)	96 785 (7.9)	1.57	1.25–1.97	< 0.001	
	7.0–7.9	104 (9.9)	69 832 (5.7)	1.16	0.89–1.52	0.3	
	≥ 8.0	138 (13.1)	148 241 (12.1)	0.76	0.61–0.98	0.04	
	Unrecorded	169 (16.0)	481 476 (39.3)	0.29	0.23–0.36	< 0.001	
Age at the end of the study period (December 31, 2019 [years])							< 0.001
	4.5–9.0	159 (15.1)	354 062 (28.9)	Base			
	≤ 4.5	19 (1.8)	505 979 (41.3)	0.09	0.06–0.15	< 0.001	
	9.0–13.5	379 (36.0)	192 345 (15.7)	5.16	4.25–6.26	< 0.001	
	13.5–18.0	194 (18.4)	123 738 (10.1)	9.38	7.76–11.34	< 0.001	
	≥ 18.0	13 (1.2)	24 503 (2.0)	4.24	2.99–6.00	< 0.001	
	Unrecorded	289 (27.5)	24 503 (2.0)	0.51	0.21–1.25	0.10	
Sex							< 0.001
	Female	380 (36.1)	615 016 (50.2)	Base			
	Male	665 (63.2)	596 638 (48.7)	1.81	1.60–2.05	< 0.001	
	Unrecorded	8 (0.8)	13 476 (1.1)	0.92	0.46–1.86	0.82	
Neuter status							< 0.001
	Neutered	853 (81.0)	747 329 (61.0)	Base			
	Entire	192 (18.2)	464 324 (37.9)	0.36	0.31–0.42	< 0.001	
	Unrecorded	8 (0.8)	13 477 (1.1)	0.50	0.25–1.00	0.05	

^a^ Population size differed for purebred status and breed variable status; data extracted from patient electronic health records for cats under UK primary veterinary care during 2019 and for cats with an incident (2019) diagnosis of diabetes mellitus.

### 3.2 Risk Factor Analysis

Univariable logistic regression modeling (Table 1) showed all six variables analyzed were moderately associated with a diagnosis of DM (*p* < 0.2) and were carried forward to the multivariable model (Table 2).

**TABLE 2:** Final breed‐focused multivariable logistic regression results in cats in the VetCompass database with an incident (2019) diagnosis of DM mellitus while under UK primary veterinary care (*n* = 1053) and compared to cats under UK primary veterinary care (2019) without a diagnosis of DM (*n* = 1 254 077). The results shown here for purebred status were from a multivariable model that accounted for four other variables: age, sex, adult body weight, and neuter status.

Variables	Category	Odds ratio	95% CI*	*p*
Breed				
	Cross Breed	Base		
	Burmilla	8.30	2.59–26.6	< 0.001*
	Burmese	2.07	1.29–3.31	0.003*
	Abyssinian	1.78	0.44–7.21	0.42
	Maine Coon	1.29	0.67–2.50	0.44
	Siberian	1.25	0.17–8.92	0.83
	Russian Blue	1.05	0.26–4.24	0.94
	Norwegian Forest Cat	0.92	0.23–3.69	0.90
	Other purebreds	0.88	0.36–2.13	0.78
	Siamese	0.62	0.28–1.38	0.24
	British Short Hair	0.59	0.35–1.00	0.05
	Unrecorded	0.51	0.28–0.93	0.03
	Persian	0.38	0.14–1.02	0.06
	Bengal	0.24	0.06–0.98	0.05*
	Ragdoll	0.11	0.02–0.80	0.03*
	Sphinx	—	—	—
Age at the end of the study period (December 31, 2019 [years])				
	4.5–9.0	Base		
	≤ 4.5	0.12	0.08–0.20	< 0.001*
	9.0–13.5	5.11	4.19–6.24	< 0.001*
	13.5–18.0	9.48	7.79–11.53	< 0.001*
	≥ 18.0	4.77	3.33–6.83	< 0.001*
	Unrecorded	1.12	0.45–2.78	0.81
Sex				
	Female	Base		
	Male	1.97	1.73–2.24	< 0.001*
	Unrecorded	1.77	0.87–3.59	0.12
Adult (≥ 9 month bodyweight [kg])				
	4.0–4.9	Base		
	≤ 2.9	0.57	0.41–0.80	0.001*
	3.0–3.9	0.84	0.64–1.10	0.20
	5.0–5.9	1.14	0.91–1.43	0.27
	6.0–6.9	1.39	1.11–1.75	0.004*
	7.0–7.9	1.10	0.84–1.44	0.50
	≥ 8.0	0.86	0.68–1.09	0.22
	Unrecorded	0.47	0.38–0.59	< 0.001
Neuter status				
	Neutered	Base		
	Entire	0.85	0.72–1.00	0.05*
	Unrecorded	1.77	0.87–3.59	0.12

*refers to statistically significant results.

The final multivariable model, which used all cats in the case control study, retained five variables: breed, age, sex, adult body weight, and neuter status (Table 2). The final multivariable model showed no evidence of poor model fit (Hosmer‐Lemeshow test statistic: *p* = 0.36).

Compared with crossbreeds, increased odds of a DM diagnosis were observed in the Burmese (odds ratio [OR], 2.07; 95% CI, 1.29–3.31; *p* = 0.03) and Burmilla (OR, 8.30; 95% CI, 2.59–26.62; *p* < 0.001). The Bengal (OR, 0.24; 95% CI, 0.06–0.98; *p* = 0.05) and Ragdoll (OR, 0.11; 95% CI, 0.02–0.80; *p* = 0.03) showed decreased odds of a DM diagnosis. The odds of DM increased with age when > 9.0 years old. Male cats showed 1.97 (95% CI, 1.73–2.24; *p* < 0.001) times the odds, and intact cats had 0.85 (95% CI, 0.72–1.00; *p* = 0.05) times the odds of a DM diagnosis, respectively (Table 2). After replacing the breed variable in the final model, purebred cats overall showed 0.71 (95% CI, 0.56–0.90; *p* = 0.01) times the odds of a diagnosis of DM compared with crossbreeds (Table 3).

**TABLE 3:** Final purebred‐focused multivariable logistic regression results in cats in the VetCompass database with an incident (2019) diagnosis of DM while under UK primary veterinary care (*n* = 1053) and compared to cats under UK primary veterinary care (2019) without DM (*n* = 1 254 077). The results shown here for purebred status were from a multivariable model that accounted for four other variables: age, sex, adult body weight, and neuter status.

Variable	Category	Odds ratio	95% CI*	*p*
Purebred status				
	Crossbreed	Base		
	Pure	0.71	0.56–0.90	0.01*
	Unrecorded	0.93	0.43–1.99	0.85

*refers to statistically significant results.

### 3.3 Mortality

**FIGURE 1:**
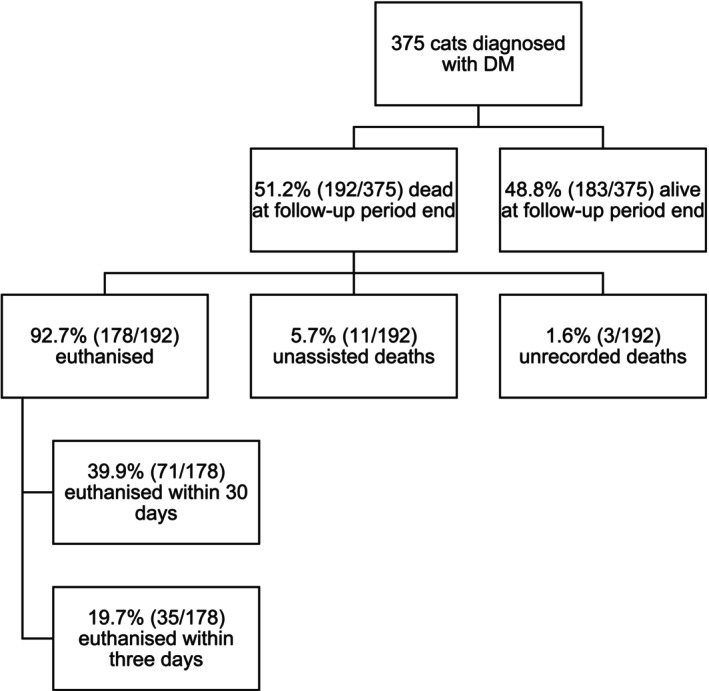
Flow diagram to show the total number of dead and alive cats after incident (2019) diagnosis of DM while under UK primary veterinary care in the VetCompass database (*n* = 375).

Overall, 192 of 375 cats (51.2%) with an incident (2019) diagnosis of DM were recorded as having died by December 31, 2021 (Figure 1). The median time to death after DM diagnosis was 68.0 days (IQR, 9.0–361.0; range, 0.0–950.0). Euthanasia accounted for 178 (92.7%) deaths and 11 (5.7%) deaths were recorded as natural (Figure 1). The type of death was unrecorded in three (1.6%) cats (Figure 1). DM was the direct cause of death in 95 (49.5%) incident (2019) DM cases, whereas DM was a contributory cause of death in 55 (28.6%) cats. Death was unrelated to DM in 21 (10.9%) cats (Figure 1).

**FIGURE 2:**
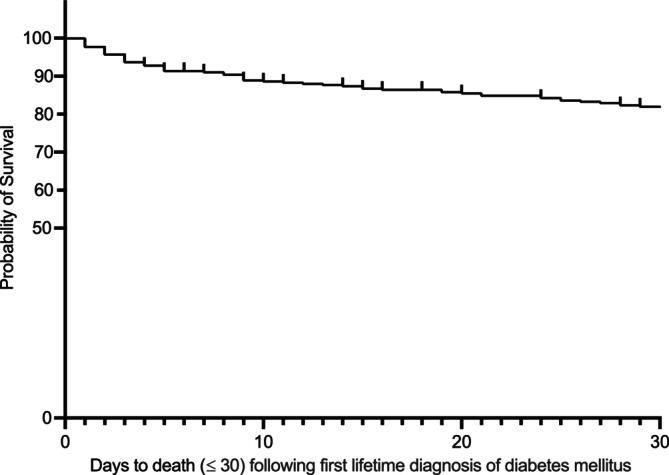
A Kaplan–Meier curve demonstrating ≤ 30‐day survival after an incident (2019) diagnosis of DM in cats while under UK primary veterinary care in the VetCompass database (*n* = 71). Vertical dashes indicate censoring of cats.

**FIGURE 3:**
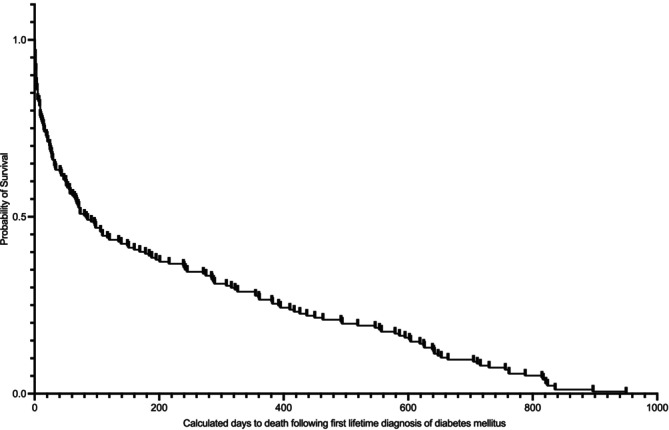
Kaplan–Meier curve demonstrating overall survival during the study follow‐up period, after an incident (2019) diagnosis of DM in cats while under UK primary veterinary care in the VetCompass database (*n* = 375). Vertical dashes indicate censoring of cats.

The ≤ 3 day, ≤ 30 day (Figures 1 and 2), and overall survival (Figures 1 and 3) were evaluated and recorded in cats with an incident (2019) DM diagnosis. The proportional mortality ≤ 3 days after an incident (2019) diagnosis of DM in cats under UK primary veterinary care was 10.0% (36/375; 95% CI, 6.90–13.15) where 97.2% (35/36) cats that died were euthanized. Of the 36 cats that died ≤ 3 days after an incident (2019) diagnosis of DM, 72.2% (26/36; 95% CI, 54.57–85.21) failed to receive any antihyperglycemic medication.

The proportional mortality ≤ 30 days after an incident (2019) diagnosis of DM was 19.7% (74/375; 95% CI, 15.90–24.20) whereas the proportional mortality related specifically to euthanasia during this same time period was 18.9% (71/375; 95% CI, 15.17–23.35). Of all cats euthanized after an incident (2019) DM diagnosis, 39.9% (71/178; 95% CI, 32.71–47.50) occurred ≤ 30 days after diagnosis.

Proportional overall risk of death during the 3‐year follow‐up period was 51.2% (192/375; CI, 46.02–56.35) whereas the overall risk for euthanasia in the same 3‐year period was 47.5% (178/375; 95% CI, 42.33–52.65).

## 4 Discussion

The 2019 DM period prevalence of 0.39% in our study is consistent with the prevalence reported in the UK among an insured population of cats [3], but lower than the prevalence reported in an earlier epidemiological study investigating DM in cats under UK primary veterinary care [2]. We, however, studied a more recent population and our results reflect a more current prevalenfunce of DM in cats. Variations in the reported prevalence may reflect intrinsic differences in populations and inherent selection bias [2, 3, 5, 9, 11].

The annual (2019) incidence risk for DM in cats under UK primary veterinary care was 0.14%, a result that is similar to a study of an insured population of Swedish cats [8].

Almost 2/3 (63.2%) of diabetic cats in our study were male. After accounting for confounding variables in the multivariable analysis, male cats had approximately 2.0 times the odds of DM compared with females. Male sex as a strong DM risk factor is consistent with the majority of previous studies [3, 8, 9, 12, 30, 31] although discordant results were published in an earlier UK epidemiological study [2]. Approximately 1/5 (18.2%) of diabetic cats under UK primary veterinary care were recorded as intact and reported as having 0.85 the odds of DM compared to neutered cats (Table 2). There were, however, several limitations to this risk factor analysis, including the young age of many intact cats in the 500 000 non‐case cat population to which prevalent diabetic cats were compared. Our study included age in the multivariable modeling to account for any confounding effects of age on the odds of a DM diagnosis. Similarly, inaccurate neuter status recording in EHR may account for the higher than anticipated prevalence of intact cats with DM. Finally, neuter status at the time of DM diagnosis was not investigated, and the links among obesity, neuter status, and DM in cats are complex and remains incompletely understood.

Diabetes mellitus in cats is considered a useful research model for type 2 DM in humans with several shared inter‐species clinical and clinicopathological features, including some risk factors such as obesity [13, 32, 33, 34, 35, 36]. Eighty‐one percent (853/1053) of prevalent DM cases occurred in cats that were recorded as neutered. Neutering is associated with the development of obesity in cats, which is an extensively researched risk factor for both DM in cats and type 2 DM in people [2, 9, 13, 14, 36, 37]. Results from our final multivariable logistic regression model determined that only cats weighing 6.0–6.9 kg retained significantly increased odds of DM (Table 2), which is largely equivalent to the median adult body weight recorded in cats 1 week on either side of an incident (2019) DM diagnosis. Nevertheless, the finding that only one adult body weight category retained significance in our study was surprising, unlike previous studies that identified higher body weight as a risk factor for DM in cats [2].

Body composition data are required to objectively assess body weight [36, 37], but such data were available in only 105 (28.3%) cats with an incident (2019) diagnosis of DM, of which over 50% of cats were described as underweight. A possible explanation for this result could be weight loss associated with uncontrolled DM [7, 10].

Increasing age is an established risk factor for type 2 DM in both humans and cats, and our data confirm this risk factor in cats [2, 8, 11]. Decreasing pancreatic β cell mass is known to occur in aging and obese cats because of amyloid accumulation, which may develop independently of an insulin‐resistant state [38]. This information offers primary care veterinarians additional information when formulating health care plans for aging cats and informing their owners of disease risks associated with increasing age.

We identified significantly decreased odds of DM in purebred cats overall compared with crossbreed cats, and supporting data that Burmese cats have increased odds of DM when compared with other purebred cats [2, 3, 6, 8, 9, 15, 16, 39]. A specific explanation why purebred cats had decreased risk of DM compared with crossbreed cats was not apparent, and would most likely require additional focus on environmental risk factors including diet, eating habits, insurance status, and indoor‐outdoor access [9].

Metabolic profiles from European and Australian Burmese cats indicate similarities to insulin‐resistant humans, including decreased concentrations of the adipokine adiponectin [2, 8, 15, 16, 39].

Moreover, we identified a novel finding of DM predisposition in the Burmilla breed that showed 8.3 times the odds of DM compared with crossbred cats. The Burmilla has not been reported previously to have increased odds of DM, but earlier studies may have been underpowered to specifically investigate this breed [2, 5, 8]. The Burmilla originated in the 1980s from mating Persian toms to Burmese queens, and this genetic relationship to the Burmese may explain the results obtained in both purebred cats [40].

Unlike previous studies, our results did not support DM predisposition in Norwegian Forest Cats [2]. Specific evaluation of the Tonkinese was not performed in our study, despite having been previously reported as a purebred with increased odds of DM [2] because a limited number of individual Tonkinese cats under UK primary veterinary care during 2019 was identified in the VetCompass program.

Most previous risk factor analyses for DM in cats have focused on identifying predispositions. In our study, Bengals and Ragdolls were identified with decreased odds of DM compared with crossbreed cats. Earlier studies had suggested decreased risk of DM in purebred Birman cats [16], but for the same reason of underpowering as Tonkinese cats in our study, Birmans were not investigated because of the low available numbers of cats in the study.

Although we identified several purebred cat breeds with increased or decreased odds of DM, the relatively small numbers of cats in the overall study from some of these breeds should be borne in mind when interpreting the results. Wide CI for results from breeds with small numbers can be interpreted as showing high uncertainty on the precision of the result reported.

Although our study has identified high mortality rates soon after a diagnosis of DM, additional information regarding the decisions surrounding euthanasia was not extracted for the current analysis [19, 20, 21]. The 10.0% incidence of euthanasia ≤ 3 days after first DM diagnosis was high; approximately 75% of dead cats did not receive any antihyperglycemic treatment. Of the incident (2019) DM cases, more than half of these had died or were euthanized by the end of the follow‐up period, and these deaths showed a median time from diagnosis to death of 68.0 days. Given that the main reason for death in diabetic cats was euthanasia, this finding suggests that future work to generate a better understanding of the clinical, welfare, and human factors that lead to such a decision could contribute substantially to improving the welfare and longevity of cats with DM.

Studies using validated quality of life (QoL) tools for cats with DM and their owners have highlighted factors that restrict an owner's day‐to‐day life (e.g., insulin administration) are more likely to negatively influence owner perceptions of their cat's QoL [18]. Until recently, the standard clinical management for DM in cats consisted of injecting exogenous insulin [7, 10, 17, 31]. The recent UK licensing of SGLT‐2 inhibitors for the treatment of DM in cats offers the potential to simplify DM management in cats with a once‐daily, PO medication [22]. Future studies evaluating euthanasia after DM diagnosis in cats are required as a result of these newer and potentially simpler medication and management options.

Our study had some limitations. Data were not primarily recorded in the EHR for research purposes. Diagnostic results were interpreted by primary care veterinarians, and a final diagnosis of DM was not validated by an internal medicine specialist. However, we consider a diagnosis of DM easier to achieve than most other endocrinopathies, and diagnostic confirmation should be readily achievable on the basis of easily performed diagnostic tests. Some true cases of DM may have been missed if DM (or synonym) was not recorded in the EHR. Additionally, the specific date of DM diagnosis could not be confirmed in > 25% of prevalent DM cases. Our study did not investigate several previously reported risk factors associated with a diagnosis of DM in cats, such as insurance status, availability of outdoor access, meal frequency, or diet [4, 8, 9, 20]. Data regarding concurrent diseases were not evaluated during statistical analysis. Finally, although collected, data exploring the types of antihyperglycemic treatment administered to cats with DM were not reported in our epidemiological study. These data will be included in a future report.

## 5 Conclusions

Our data provide additional support for breed, male sex, neutering, increasing age, and crossbreed cats as important risk factors for DM. As well as reaffirming evidence for DM predisposition in Burmese cats, we also report the Burmilla as predisposed for DM. Additionally, we provide evidence for protection against DM in Bengals and Ragdolls.

Complex relationships exist between genotypic and phenotypic factors associated with the development of DM in cats. Additional studies are required for further exploration of the relationship between adult body weight, body composition, and neuter status in cats with DM. We believe that DM mortality rates in cats should be reviewed, based on the licensing of SGLT‐2 inhibitors in the UK, simplifying diabetic management and minimizing effects on owners' and their cats' QoL.

## Disclosure

Authors declare no off‐label use of antimicrobials.

## Ethics Statement

Approved by the Royal Veterinary College Social Science Ethical Review Board (reference number SR2018‐1652).

## Conflicts of Interest

The authors declare no conflicts of interest. Neither the Kennel Club Charitable Trust nor Agria Pet Insurance had any input in the design of the study, the collection, analysis, and interpretation of data, or in writing the manuscript.
